# Cytokine profiling: variation in immune modulation with preterm birth vs. uncomplicated term birth identifies pivotal signals in pathogenesis of preterm birth

**DOI:** 10.1515/jpm-2020-0025

**Published:** 2020-10-12

**Authors:** Jeffrey M. Denney, Edward Nelson, Pathick Wadhwa, Thaddeus Waters, Leny Mathew, Robert L. Goldenberg, Jennifer F. Culhane

**Affiliations:** Department of Obstetrics and Gynecology, Wake Forest School of Medicine, Section for Maternal-Fetal Medicine, Winston-Salem, NC, USA; Department of Obstetrics & Gynecology, Drexel University College of Medicine, Philadelphia, USA; Department of Hematology & Oncology, University of California—Irvine, School of Medicine, Irvine, USA; Department of Obstetrics & Gynecology, University of California—Irvine, School of Medicine, Irvine, USA; and Department of Obstetrics & Gynecology, Loyola University, Division of Maternal-Fetal Medicine, Maywood, USA; Department of Pediatrics, Children’s Hospital of Philadelphia, Division of Adolescent Medicine, Philadelphia, USA; and Department of Obstetrics & Gynecology, Columbia University of Physicians and Surgeons, New York, USA; Department of Pediatrics, University of Pennsylvania, School of Medicine, Division of Adolescent Medicine, Philadelphia, USA; and Yale University, School of Medicine, New Haven, USA

**Keywords:** cytokines, immune profiling, inflammation, preterm birth

## Abstract

**Objectives:**

To assess deviations in longitudinally measured cytokines with preterm birth (PTB).

**Methods:**

Prospective longitudinal study targeting 80 subjects. Phlebotomy specimens for broad panel of cytokine analysis were obtained at three time (T) intervals: first trimester (T1: 8–14 weeks’ gestation), second trimester (T2: 18–22 weeks’ gestation), and third trimester (T3: 28–32 weeks’ gestation). Important demographics and outcomes were tracked. Data were stratified and the target groups were analyzed as follows: “Uncomplicated” (delivered ≥37 weeks) or “Preterm Birth” (<37 weeks). Generalized Linear Modeling determined rate of change T1–T3 by outcome.

**Results:**

Complete data replete with phlebotomy at all three visits were obtained on 80 women. Birth outcomes were as follows: 11 Uncomplicated Term Birth (UTB), 28 PTB, 4 low birth weight (LBW), 16 OB complications (OBC), 11 current infections (IFN), and 10 mixed complications (MC=2 or more of the above). 28 PTB were compared to 11 uncomplicated term deliveries. In both groups, T helper type 1 (TH1) cytokine (IL-1β), pleiotrophic pro-inflammatory cytokine (IL-6), and counter-regulatory cytokine (IL-10) responses decreased over gestation, but rates of change in IL-1β, IL-6, and IL-10 were significantly different. Stratification of women by smoking status additionally demonstrated significant variance in immune status over the course of pregnancy

**Conclusions:**

Women delivering PTB demonstrated significant differences in cytokine trajectory over pregnancy; these data further validate key role played by immune regulation in directing pregnancy outcome. Likewise, smoking impacts longitudinal trajectory of cytokines over pregnancy.

## Introduction

Shifts in immune parameters in pregnancy have been implicated in pregnancy outcomes. A number of adverse pregnancy outcomes like preeclampsia, preterm premature rupture of membranes, and miscarriage have all been hypothesized to have a strong association with increases in systemic inflammation as demonstrated by various serum markers. A number of environmental factors (e.g., smoking, substance abuse, pollution) and health conditions (e.g., pelvic infection, periodontal disease) are known to both increase systemic inflammation and in turn increase risk for preterm delivery [1], [2], [3], [4]. While many inflammatory markers exist, cytokines are a diverse family of soluble small proteins, expressed by various cells and tissue types; these proteins act as immune mediators and the mapping corresponding expression profile may be important in predicting at risk pregnancies.

Maternal immune responses are modified during pregnancy. Prior data indicate a trend over gestation toward a dampened T helper type 1 (Th1)-associated and pleiotrophic cytokine expression; such data implicate enhanced counter regulatory cytokine expression as instrumental in decreasing inflammatory responses and, in effect, tolerance to fetal tissues [5]. Other data further support the belief that maternal immune modulation plays a vital role for both establishment of and maintenance of a viable pregnancy [6], [7].

T cells have often been the focus of many reproductive studies given their role in signaling and coordination of immune responses via cytokine signaling [8], [9], [10]. Given this role, experts have identified inflammatory insults and ability to identify inflammation as a target for the prevention of preterm birth [11]. A cytokine expression profile can be used to categorize coordinated systemic immune responses [12]. Assessing multiple cytokines is paramount to characterize the status of the immune system with regard to the Th type bias and intrinsic levels of inflammation [13]. Previously, Velez et al. reported cytokine variance in a cohort study of women delivering either term or preterm. Their data while taken from in a singular time point in labor from amniotic fluid added to the growing body of evidence that cytokine signaling is divergent from normal term pregnancy when women spontaneously deliver preterm.[14]. Our group has previously validated assessment of a broad panel of cytokines for immune profiling.

While prior data on cytokine response in pregnancy suggest immune modulation plays an important role in normal pregnancy [5], complete characterization and understanding of immune modulation in women achieving uncomplicated term deliveries vs. preterm delivery remains incomplete [9], [10]. The bulk of existing studies are limited by cross-sectional design.

Moreover, the trajectory of cytokine expression over the course of pregnancy in term vs. preterm is not well characterized. A number of questions surround whether cytokine profiles differ at time of preterm delivery only or if trajectory from early and mid-trimesters vary as well [14]. Such gaps underpinned the impetus to perform an investigation aimed at describing immune state as pregnancy develops in both women that ultimately carry to term vs. those that spontaneously deliver prior to completing 37 weeks gestational age [15]. Likewise, our group had concerns regarding lack of knowledge of what is occurring prior to labor beginning in terms of inflammatory states leading up to labor in both groups.

A number of biological fluids have been sampled for assessing inflammation as it pertains to preterm birth; these have included amniotic fluid, cervical secretions, urine, saliva, serum, plasma, vaginal secretions, and periodontal fluid. Each type of fluid has both strengths and limitations with respect to knowledge that can be gained. Preterm birth is an endpoint in many respects that which multiple pathways may lead to triggering inflammation, upregulation of oxytocin receptors and downregulation of progesterone receptors whether the inflammation originates from the gingiva or the vagina for example. Arguably, the best clinical marker for the prediction of spontaneous preterm birth to date has been fetal fibronectin in the cervix and/or vagina [16].

However, cervicovaginal fluids are only one pathway that leads to preterm birth. Evidence continues to mount that periodontal disease creates local inflammation in the oral cavity that spreads systemically and affects other organ systems. Such harm is not limited to increased incidence of cardiovascular disease and myocardial infarction but joint periodontal and obstetrical investigators have demonstrated a strong association of periodontal disease when untreated leads to increased systemic inflammation that in turn seeds the cervix with inflammatory changes leading to early shortening and in turn preterm delivery. Like cervicovaginal lavages, saliva is the ultrafiltrate of the plasma and facilitates assessment of a number of inflammatory markers from C-reactive protein (CRP) to cytokines. However, both cervicovaginal and saliva fluid samples are limited by patient activity (e.g., oral lesions, vaginal abrasions, vaginal intercourse, use of oral and vaginal hygiene products/soaps/rinses and may also allow maternal serum proteins to exude more readily or be degraded by chemicals for hygiene in the vagina or mouth, thus either falsely elevating or depressing the levels. These behaviors are difficult to limit and control. Infection and inflammation may both be present but notably inflammation may arise systemically and present to the myometrium and cervix in absence of infection as in psychological stress or from metabolic and/or cardiovascular disease processes. Serum assessment can, on the other hand, be collected in absentia of such environmental insults and be collected with little to no risk to the pregnancy. Phlebotomy collection is typically timed every trimester regardless for routine standard assessments for the patient’s prenatal care to not add much in the way of added visits or examinations.

Multiple environmental and clinical insults may arise in gestation and elicit inflammatory stress response whether the entry point be urogenital infection, periodontal disease, psychological stress due to anxiety or depression affecting cortisol and in effect inflammatory milieu, or even adipose load raising systemic inflammation that ultimately presents to the uteroplactental interface and may affect labor onset [15], [16], [17], [18]. The women in our inner city population have a number of risk factors for inflammation with cervicovaginal infection/colonization representing one portal presenting at the nexus to signal/trigger labor onset whether term or preterm [18], [19]. Cortisol releasing hormone (CRH) and hypothalamic-pituitary-adrenal axis (HPA axis) hormones are known to influence inflammatory response to both infection and psychosomatic stress [20], [21]. Such complex interactions and modification of systemic inflammatory states have not only been implicated but established as playing a role in preterm birth [22], [23].

Our aim was to compare cytokine profiles in pregnancies ending in either uncomplicated term delivery or spontaneous preterm delivery. Our approach of obtaining a broad-panel of cytokines at three gestational time-points intended to identify immune variations of etiologic importance with respect to term vs. preterm delivery.

## Materials and methods

### Study design

This study was part of the Philadelphia Collaborate Preterm Prevention Project sponsored by The Children’s Hospital of Philadelphia (CHOP) and Drexel University. Institutional Review Board (IRB) approval was obtained from both universities (CHOP IRB 09-007191, date of approval 12/28/2009 and Drexel IRB 40568, date of approval 9/10/01, renewal 9/10/09). We initiated a prospective, longitudinal investigation of immune and neuroendocrine parameters associated with stress, infection, and preterm birth.

### Eligibility and enrollment

For this study, all women attending their first prenatal visit at a hospital-based clinic in Philadelphia, Pennsylvania between March 2001 and November 2002 were screened for eligibility. Women were considered eligible for participation if they were, (1) English or Spanish speaking, (2) had a singleton intrauterine pregnancy, (3) <15 weeks’ gestation, and (4) ≥17 years of age.

Conversely, women were excluded from participating if they had any immunologic disorder (e.g., Human Immunodeficiency Virus (HIV)/Acquired Immunodeficiency Syndrome (AIDS), Systemic Lupus Erythematosis (SLE), sarcoidosis, taken systemic corticosteroids six months prior to enrollment, or taken antibiotics for infection in the 15 days prior to enrollment. All eligible women were approached for consent, and those who provided written informed consent were enrolled. Women became ineligible at the second or third trimester visits if they were no longer pregnant, developed an excluding medical condition, or if they moved out-of-state and could no longer take part in the study.

### Data collection

Trained interviewers collected demographics, medical history, behavioral history, and psychosocial data from study participants upon enrollment. Study participants were followed at regularly scheduled prenatal appointments with updated data. Phlebotomy specimens were obtained at three intervals: first trimester (T1: 8–14 weeks’ gestation), second trimester (T2: 18–22 weeks’ gestation), and third trimester (T3: 28–32 weeks’ gestation). These intervals were carefully selected to accomplish the following: (1) avoid early immune shifts and variation during the initial phase of implantation; (2) ensure enrollment after well-established viability and diagnosis of singleton intrauterine pregnancy; (3) assess phlebotomy and urine at times that coincided with usual standard of care visits; (4) be representative time points within all three trimesters in well-dated pregnancies; and (5) avoid drop out and bias due to preterm delivery to facilitate comparison across the two groups that would either deliver preterm or term.

Upon enrollment, all women completed a face-to-face interview with trained study personnel to assess: (1) socio-demographic history, (2) psychosocial history, (3) health behavior history, and (4) medical history. Maternal variables abstracted by research staff were those having pertinent effects on preterm birth; these included the following: age, race, substance abuse (e.g., smoking, alcohol, illicit/recreational drug use), education level, and income. Alcohol use was qualified by patient self-report during the face-to-face interview. Criteria for being classified as positive for pregnancy complicated by illicit/recreational drug use was qualified by a positive urine drug screen or positive patient report of drug use during pregnancy of any one or more of the following: cocaine, marijuana, phencyclidine (PCP), amphetamines, opiates, benzodiazepines, barbiturates, methadone, propoxyphene, and/or quaaludes. Likewise, as part of the consent process for the study, our physician panel had the opportunity to review all prenatal care records, including outpatient clinic visits, hospitalization records, delivery records, neonatal records during initial hospitalization, and postpartum care to review for any positive report or test result of any substance use, including tobacco, alcohol and all illicit/recreationally used drugs or prescribed substance dependency. These results were abstracted into the data sheets for each patient accordingly for demographics and outcomes as either positive/negative for substance use or likewise either positive/negative for smoking.

Anthropometric measurements (e.g., maternal height and weight) were obtained. All participants had their body mass index (BMI) calculated. Standard cutoffs for BMI were used as follows: overweight (>25), obese (>30), morbidly obese (>35).

Medical records were reviewed to obtain and verify antepartum, intrapartum, and post-partum information about the study participant’s demographics, history and medical conditions as well as documentation of a live birth from available medical records. To ensure accuracy of the medical record information, medical records were abstracted by trained personnel and collectively verified by a physician panel. Obstetrical complications included abruption/previa, intrauterine growth restriction (IUGR) as demonstrated by low birth weight (LBW) on Fenton index chart, gestational hypertension (GHTN)/pre-eclampsia, or fetal distress. Abruption was the coded indication for delivery when maternal hemorrhage suggestive of abruption was the documented reason for delivery by cesarean. When placenta previa was documented in the chart as due to ultrasound findings, the indication for delivery by cesarean was coded as previa. Fetal distress was defined as non-reassuring fetal heart tracing (NRFHT), repetitive fetal heart rate decelerations prompting an indicated delivery, or if a Category III fetal heart tracing was noted. Low birth weight was defined by birth weight less than the 10th percentile for the assigned gestational age per Fenton growth chart. Gestational hypertension (GHTN) was defined as at least two maternal blood pressures ≥140/90 in the absence of proteinuria. Criteria for pre-eclampsia were defined as at least two maternal blood pressures over a 4 h interval of ≥140/90 with proteinuria (≥1 + protein on 2 separate urinalyses or ≥300 mg protein on 24 h urine collection. Patients who progress to either severe pre-eclampsia or severe GHTN were coded as an indicated delivery for “pre-eclampsia” or “GHTN,” respectively. All of these were excluded from this analysis as the purpose of this paper was to compare immune profile of uncomplicated term delivery to otherwise uncomplicated spontaneous preterm birth.

When chorioamnionitis or intraamniotic infection (IAI) was the indication for delivery, we examined six individual variables: (1) maximum recorded intrapartum temperature, (2) maternal leukocytosis (≥20,000 mL white blood cell count (WBCs), (3) maternal tachycardia (maternal heart rate >100 beats per minute (bpm) for 20 min), (4) fetal tachycardia (fetal heart rate >160 bpm for ≥20 min), (5) malodorous amniotic fluid (when documented by labor and delivery nurse/physician), and (6) uterine tenderness (when documented by examining physician in the medical record). Those identified as having infection (e.g., pyelonephritis or chorioamnionitis at time of delivery or identified as such during their pregnancy were excluded from the analysis).

### Immunologic assays

At each of three time points, phlebotomy samples were collected by standard antecubital venipuncture with vacutainer collection in sodium heparin anticoagulant, for *in vitro* cytokine release assays. For the cytokine release assay, the sample of anti-coagulated maternal blood was collected and transported on ice within 30 min to the on-site laboratory for processing as follows: the cytokine release analysis was performed on incubated supernatants from stimulated whole blood. For the cytokine release assay, 24-well plates were prepared in advance with 1.5 mL of media consisting of Roswell Park Memorial Institute’s RPMI 1640 (Invitrogen, Carlsbad, CA) containing 10% fetal bovine serum (Invitrogen, Carlsbad, CA) and penicillin/streptomycin (Invitrogen, Carlsbad, CA) at 1:100 dilution added to individual wells. Plates were stored at −20 °C, thawed and brought to room temperature immediately before use. Heparin anti-coagulated blood samples were distributed, 0.5 mL of whole blood, into four individual wells of the previously prepared 24-well plate containing one of the three conditions: (1) supplemented with phytohemagglutinin (PHA) (Sigma, St. Louis, MO) at a final concentration of 5 μg/mL, (2) supplemented with lipopolysaccharide (LPS) −2 (Sigma, St. Louis, MO) at a final concentration of 1 μg/mL, or (3) not supplemented as the control.

All plates were incubated for 24 h at 37 °C in 5% humidified CO_2_. At the conclusion of this period, plates were placed on ice and the contents of the four wells for each condition were transferred to 15 mL conical tubes. Samples were centrifuged at 4 °C for 10 min at 300 × g followed by distribution of the supernatant into 500 µL aliquots that were immediately frozen at −80 °C and maintained frozen until analysis in triplicate by Luminex-100 MAP^®^ methods using commercially available kits (Linco Research Inc. St. Charles, Missouri) according to manufacturer’s instructions and as previously reported [12].

Our group extensively researched existing data on immune variation in pregnancy as it relates to preterm birth and inflammation as opposed to the immune state demonstrated by women delivering at term. As previously mentioned, most of these data were derived from cross-sectional study design, making our longitudinal design essential for understanding how trajectory of cytokine milieu over the course of these gestations [23], [24], [25]. We also needed to represent the overall immune response in a broad-panel of cytokines that are involved in (1) monocyte/macrophage mediated responses (T cell helper type 1 (Th1) pathway); (2) antibody-mediated that promote mast cell and eosinophil production (T cell helper type 2 (Th2) pathway); and (3) cytokines exerting their regulatory functions in a Th1/Th2-unrestricted fashion and in effect regulate T-helper cell development and responses in an over-arching regulatory role. Utilizing these classic frameworks for selection of cytokine signals that would compose an image or profile of an individuals’ immune state at selected time intervals in gestation, we additionally utilized existing evidence for important variations in representative cytokines in preterm birth. A Th1/Th2 imbalance remains as a paradigm for specific disease related immune responses albeit many studies contradict one another, which may be due to their limitation of cross-sectional design. Rather than arbitrary cut-points in cytokine concentration for elevation or not, the key to understanding cytokine profiles as they relate to pregnancy outcome for an individual may lie in rate change in inflammatory status over gestation within individuals as baseline inflammatory states and cytokine baseline levels may vary from person to person.

We selected the following cytokines for analysis: IFN-γ, the prototypical Th1-associated cytokine; IL-4, a Th2-associated cytokine; the less specific pro-inflammatory cytokines TNF-α, IL-10, and IL-6; and IL-10, a predominant counter-regulatory cytokine associated with suppression of T cell maturation. An extensive literature search and experience by the investigators led to selection of these cytokines as representative for the Th1-associated, Th2-associated, pro-inflammatory, and counter-regulatory cytokine assays to represent the profile or inflammatory status of each of the subjects and by birth outcome stratification. A power calculation indicated that assuming a 15% preterm birth rate in our higher risk inner city population that 30 women would be needed in total to detect a 30% variance in cytokine trajectory between preterm and term birth outcomes. Knowing that additional pregnancy outcomes in the targeted sample would inevitable occur in those completing the three phlebotomies.

We employed two primary stimuli: (1) LPS to activate CD14 & TLR-4 expressing cells—evokes TH2 response by monocytes, and (2) PHA to activate T lymphocytes—evokes TH1 response. Together, these stimuli provided measures of induced cytokine expression for monocytes, components of the innate arm of the immune system, and for lymphocytes, integral elements of the adaptive arm of the immune system. Notably, the utilization of a mitogen has limitation as it does not precisely replicate an antigen specific response (i.e., *in vivo* interactions between monocytes/dendritic cells and T cells). However, mitogen use has been validated and is a widely-accepted approach for the study of the immune response [12], [14]. Cytokine expression was evaluated at 24 h to limit the effect of secondary induction of cytokine byproducts derived from the primary stimulus.

### Smoking status

Smoking status was assessed in enrolled subjects at each time point. Status was based on patient report and confirmed by “nicometer” utilizing measurement of urine cotinine at the same visit as each of the three phlebotomy collections to control for smoking influence on inflammatory state at each visit. The urine cotinine assay is a validated tool to assess smoking status in a “positive” or “negative” result format. An interaction term of group with gestational age was used to compare rates of change over gestation by smoking status.

### Statistical analysis

Data were stratified into two groups based upon pregnancy outcomes: (1) uncomplicated term birth (UTB): delivery at term (≥37 weeks) of normal birth weight neonate with no identified medical complications over the course of gestation; or, (2) preterm birth (PTB)--delivery <37 weeks’ gestational age with no other identified medical/obstetrical complications over the course of gestation.

We determined the rate of change for each cytokine from T1 to T3 using Generalized Linear Models (GLM with gamma distribution and log link). Adjustment for clustering was made using robust standard errors via the Huber-White sandwich estimator. An interaction term of group with gestational age was used to compare the rates of change by pregnancy outcome. Differences were defined as significant with a p-value <0.05.

For these analyses, we included all participants with complete immune data for their first three study visits and available medical record data providing documentation of a live birth. Replicate cytokine determinations were evaluated for potential outliers within the triplicate values for each sample using the Grubbs formula at the 5% critical Z-value [22]. Mean determinations for replicates from each sample were used for subsequent analysis.

To assess immune modulation during pregnancy**,** we fit a regression model to assess the trajectory of each cytokine throughout pregnancy. For all regression analyses, we used GLM with the gamma family of distributions and a log link [10]. Such models accounted for the distribution of the data. Since the cytokine values were measured repeatedly over time, and thus correlated for each subject, we calculated robust standard errors using the Huber-White sandwich estimator [10]. Statistical significance was defined at p<0.05.

## Results

A total of 80 women that met inclusion criteria had complete data as well as completed the three phlebotomy and urine samples; this longitudinal study of broad cytokine panels over the three trimesters in gestation comparing preterm birth vs. term birth is the largest of its kind to date. Notably, during the screening and enrollment process women became ineligible at the second or third trimester visits upon learning the following: (1) no longer pregnant (e.g., abortion (n=12), a stillbirth (n=2), or a miscarriage (n=21)); (2) onset of excluding medical condition (n=1), or (3) they moved out-of-state and could no longer take part in the study (n=2).

### Sociodemographic characteristics

Our cohort of 39 of 80 women with either uncomplicated spontaneous preterm delivery or uncomplicated term delivery demonstrated a mean age of 23.3 years and were predominately African-American (92.3%), multiparous (53.8%), unmarried (75%), and most did not attain at least a high school diploma (77.5%). Demographics between groups — preterm birth and term delivery — did vary by birth etiology amongst those who were both enrolled and had a live birth (Table 1). Namely, women in the preterm birth group were 28/28 nonhispanic black vs. 8/11 uncomplicated term deliveries (p=0.04 by ANOVA). Marriage or living as married demonstrated a reduction in odds of having preterm delivery (8/11 UTB vs. 7/28 spontaneous preterm birth; p<0.01; OR 0.12 95% CI 0.03, 0.61). Age, parity, income, substance use (recreational/illicit drug use, namely one or more positive of any of the following: cocaine, marijuana, PCP, amphetamines, opiates, benzodiazepines, barbiturates, methadone, propoxyphene, and quaaludes), and BMI were similar between groups (Table 1).

**Table 1:** Sociodemographic characteristics of sample stratified by birth etiology.

	Uncomplicated n=11	PTB n=28	p-Value
Age, years, median (SD)	23.6 (7.5)	23.2 (8.7)	0.91
Race/Ethnicity, %			
Non-hispanic white	1/11 (9.1)	0/28 (0)	**0.04** (ANOVA)
Hispanic/Latina	2/11 (18.2)	0/28 (0)	
Nonhispanic black	**8/11 (80.1)**	**28/28 (100)**	
Other	0/11(0)	0/28 (0)	
Substance use, %	7/11 (63.6)	18/28 (64.3)	0.96
Education, %			
Did not finish high school	3/11 (27.3)	9/28 (32.1)	0.6 (ANOVA)
High School/GED	5/11 (45.5)	14/28 (50.0)	
Continued post high school	3/11 (27.3)	5/28 (17.9)	
**Married/living as married**	**8/11 (72.7)**	**7/28 (25)**	**<0.01**
Annual income, dolars (mean, SD)	10,745.4 (97.2)	10,429.8 (124.3)	0.83
Nulliparous, %	5/11 (45.5)	13/28 (46.4)	0.95
BMI (mean, SD)	26.3 (9.4)	27.9 (7.1)	0.8

### Outcome stratification and cytokine assays

**Figure 1:**
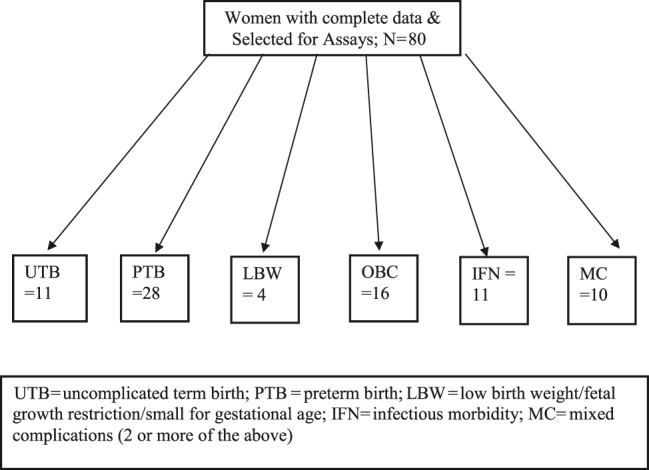
Cytokine cohort flowsheet.

Eighty women with complete data had the 54 assays run. Birth outcomes were as follows: 11 UTB, 28 PTB, 4 LBW, 16 OB complications (OBC), 11 current infections (IFN), and 10 mixed complications (MC=2 or more of the above). Per our planned analysis, 28 women with PTB were compared to 11 women with UTB; (see Table 1). We evaluated replicate cytokine determinations for potential outliers in each condition and that we utilized mean determinations for replicates from each sample in the analysis to produce the results. Notably, we performed our analysis with and without the outliers. No changes in statistical significance were noted when removing outliers from the analysis, indicating that our analysis and data sets were robust (Figure 1).

**Figure 2:**
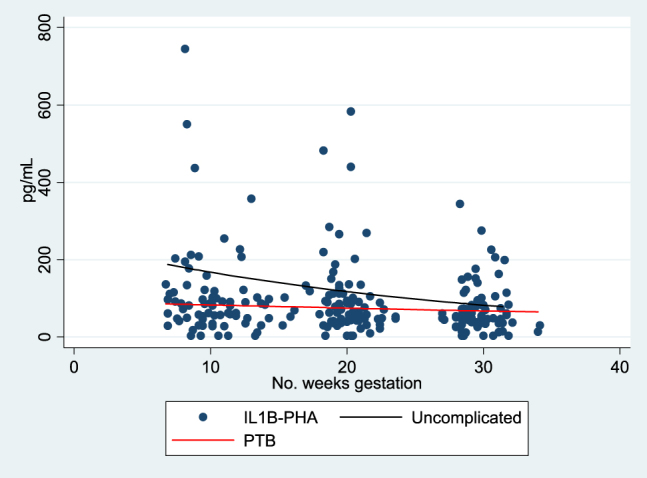
IL 1β-PHA trajectory of expression over gestation.

**Figure 3:**
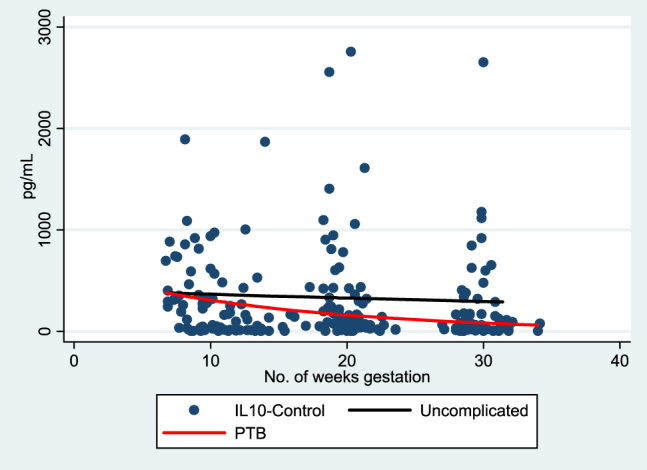
IL-10-control trajectory of expression over gestation.

**Figure 4:**
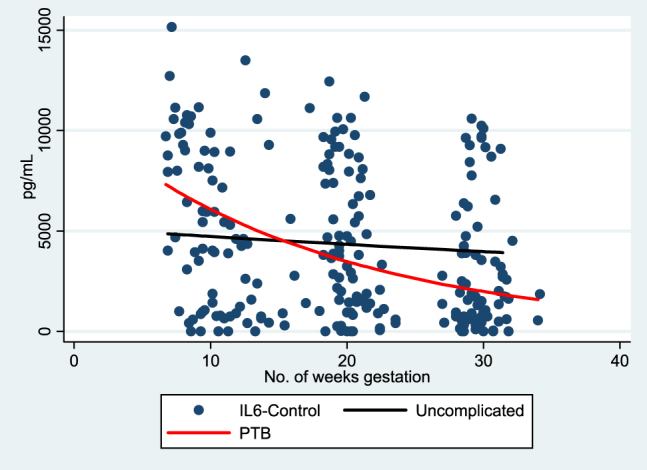
IL-6-control trajectory of expression over gestation.

In both groups, TH1 cytokine (IL-1β), pleiotrophic pro-inflammatory (IL-6), and counter-regulatory cytokine (IL-10) responses decreased over the course of gestation. However, the rates of change in IL-1β, IL-6, and IL-10 were significantly different (Table 2). The rates of change for IL-1β to PHA stimulation demonstrated more suppression (more negative slope) in uncomplicated term deliveries when compared to the otherwise uncomplicated preterm deliveries; this is illustrated in Figure 2. IL-6 showed very little change in expression over gestation in the uncomplicated term deliveries as opposed to the decrease in IL-6 demonstrated by the preterm delivery group (Figure 3). IL-10 expression decreased over gestation significantly in the preterm birth group when compared to the uncomplicated term delivery group as seen in Figure 4. For all other measured cytokines, trajectories did not vary significantly between the groups.

**Table 2:** Coefficients and p-values for cytokines differing by smoking.

Cytokine	Non-smokers	Smokers	p-Value of interaction term
IL-10—control	−0.19	−0.064	0.045
TNFα—control	−0.016	−0.049	0.040

### Data stratified by smoking status

**Figure 5:**
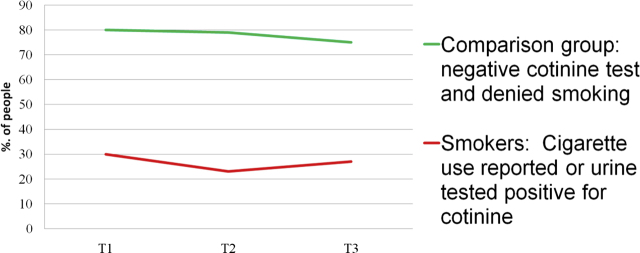
Incidence of smoking by timepoint in gestation (whole cohort of 80 women).

At T3, among the self-reported smokers (n=7 of 39 or 17.9%), 6 (85.7%) had a positive Cotinine test. Among the people with positive cotinine test (6/39), 4/6 (75%) reported smoking. Given small numbers of smoking patients within the cohort of 39 (11 UTB and 28 uncomplicated spontaneous preterm birth), we additionally looked at data on smoking for the entire group of 80 women with complete data on smoking. Figure 5 demonstrates the incidence of smoking (by % “negative” and % “positive”) over the three time points. “Negative” was defined as having both a negative cotinine test and denied smoking at each time point. “Positive” was assigned for use reported and/or urine cotinine positive. At T3, among the 14 self-reported smokers, 12 (85.71%) had a positive Cotinine test whereas among the people with positive cotinine test, only 52.17% reported smoking. Self-report of smoking seems to be under reported in this sample as measured by a cotinine test.

There were significant differences in cytokine expression by smoking status demonstrated by pleiotrophic pro-inflammatory (TNF-α) and counter-regulatory cytokine class responses (IL-10). Decrease in TNF-α found in smokers may contribute to the mechanism behind previously reported findings that smoking has a protective effect on the incidence of hypertensive disorders of pregnancy, namely that of preeclampsia (i.e., TNF-α elevation has been reported in pre-eclamptics vs. normal controls). An associated decrease in IL-10 with smoking in this analysis is consistent with previous reports on IL-10 decrease having association with both preterm birth and smoking. Table 2 illustrates the coefficients and p-values for cytokines differing by smoking. Table 3 illustrates the median values of cytokines differing by smoking status. There were no significant differences by smoking status in TH1 (IL-1β, IFN-γ) or TH2 (IL-4) class responses.

**Table 3:** Median values of cytokines differing by smoking status.

Cytokine	Non-smokers median (range)	Smokers median (range)
IL-10—control-T1	256.2 (3.2, 1894.4)	163.5 (3.2, 1091.9)
IL-10—control-T2	88.0 (3.2, 2757.8)	76.3 (3.2, 1405.3)
IL-10—control-T3	71.8 (3.2, 2655.2)	34.9 (3.2, 844.8)
TNFα—control-T1	111.2 (3.2, 809.6)	76.1 (3.2, 878.1)
TNFα—control-T2	61.8 (3.2, 662.6)	45.5 (3.2, 1076.2)
TNFα—control-T3	51 (3.2, 475.3)	39.7 (3.2, 404.1)

## Discussion

Our data indicate there are critical variations in cytokine networks leading to preterm delivery. The observation of a temporal alteration in IL-1β, IL-10, and IL-6 accentuates the notion that preterm labor is a disruption of a delicate balance essential to achieve delivery at term.

Our data show that women with either preterm birth or term delivery demonstrate similar IL-1β expression over gestation with the exception of the TH1 response. Women who ultimately delivered at term exhibited increasingly more suppression of their IL-1β expression in response to TH1 mitogen from their baseline response as gestation progressed. On the contrary, this immune modulation was not exhibited by those who ultimately delivered preterm (less negative slope from T1 to T3). Accordingly, progesterone mediated activation of membrane progesterone receptors inhibit IL-1β gene activity in prior reports. Progesterone supplementation for those with history of preterm birth is now widely used to prevent subsequent preterm delivery [26]. Our data showing lack of IL-1β suppression in the preterm deliveries suggest intrinsic cytokine variances underpin the effectiveness of the progesterone supplementation. Prior data has demonstrated progesterone withdrawal to have effects on cells with progesterone receptors, leading to cytokine upregulation, increase in local inflammation and enzymes responsible for tissue breakdown as well as vasoconstriction. Cytokine release within the myometrium has been shown to effect matrix metalloproteinase activity that has been demonstrated to lead to cervical modeling with decrease in cervical stroma and in effect cervical shortening as well as weakening of the amniotic and chorionic membranes thereby priming the gestation for labor whether preterm or term [27], [28]

Our finding of IL-10 decrease over gestation in those delivering preterm is likewise interesting. We previously found IL-10 to demonstrate an increase over normal gestation [11]. The observed deviation or reversal in trajectory by those ultimately delivering preterm suggests inadequate suppression of inflammatory responses leads to preterm birth. The IL-10 expression in our preterm patients showed lower initial levels as well as more pronounced decrease as these patients progressed into the second and third trimesters when compared to the term deliveries. Herrera-Munoz also found decreased IL-10 in the serum of women with threatened preterm labor [27]. Cecati et al. demonstrated that the IL-10 receptors were downregulated in the endometrium in a group of women recently delivering preterm when compared to non-pregnant women (experiencing term birth) by endometrial biopsy [28]. Hence, the demonstration of IL-10 decrease in our cohort of preterm deliveries again points toward IL-10’s importance in achieving term gestational age.

Preterm deliveries demonstrated a marked fall in IL-6 relative to that of the patients who ultimately achieved delivery at term. The increased negative slope across gestation with respect to both IL-6 and IL-10 is particularly interesting. IL-6 functions as both inflammatory and anti-inflammatory per a large accumulation of conflicting data. IL-6 may serve as an inhibitor of inflammatory pathways and when seen to increase or decrease in concert with IL-10, this appears to be an important coordination by the systemic cytokine network within the maternal circulation. In our data, the simultaneous decrease in both IL-6 and IL-10 is found in association with the adverse outcome of early delivery. Said another way, the inability to maintain adequate IL-6 and IL-10 concentrations appears to lower the threshold for entering the pathway of preterm labor.

Our data on IL-6 placed in context of prior data on both IL-6 and progesterone receptor polymorphisms continue to indicate intricate interactions between cytokine networks and hormonal levels. Such cytokine/hormone balance likely exists prior to the onset of labor whereby there are key tipping points that lend to immune-mediated activation of metalloproteinases and prostaglandin production for cervical ripening, along with up-regulation of oxytocin receptors, and donw-regulation of mPR (membrane progesterone receptors) to disengage maternal efforts in continuation of the pregnancy to more advanced gestational age. Given data supporting the role of the immune shift away from the proinflammatory pathway in those achieving term delivery vs. inadequate shift in those experiencing preterm delivery, our study continues to add to the mounting evidence for TH-2/antiinflammatory cytokine signaling is important to achieve an uncomplicated term delivery. TH-1 cytokines have been proven to activate macrophages. Many of the women with decreasing ability to maintain TH2 cytokine signaling may have underlying chronic infection, thus predisposing them to so-called macrophage priming and hyperresponsiveness to minor inflammatory stimuli, leading to a lower threshold to enter the labor pathway at increasing early gestational ages.

To date there are limitations to impact of smoking of inflammatory states during the course of pregnancy as most data are cross-sectional. Conversely, our data demonstrate the impact of smoking on inflammatory states over the course of the three trimesters in pregnancy longitudinally to measure impact of smoking on the trajectory of cytokine expression during pregnancy. Our data show significant differences in cytokine expression by smoking status demonstrated by pleiotrophic pro-inflammatory (TNF-α) and counter-regulatory cytokine class responses (IL-10). Decrease in TNF-α found in smokers may contribute to the mechanism behind previously reported findings that smoking has a protective effect on the incidence of PIH/pre-eclampsia (i.e., TNF-α elevation has been reported in pre-eclamptics vs. normal controls). An associated decrease in IL-10 with smoking in this analysis is consistent with previous reports on IL-10 decrease having association with both preterm birth and smoking. Our demonstration that smoking decreases IL-10 is critical as decrease in IL-10 has previously been associated with pregnancy loss and preterm birth. As prior investigations have demonstrated, IL-10 is a key counter-regulatory cytokine to suppress inflammatory response and is thought to be a key player in the immune shift to facilitate tolerance of the pregnancy and in effect advancement to term pregnancy with a mature fetus prior to onset of inflammatory cascades that ultimately trigger labor.

Likewise, smoking has long been associated with risk for both miscarriage and preterm premature rupture of membranes. Our finding implicates inflammatory alteration rooted in decrease of IL-10 when smoking during pregnancy as biologically causative in preterm birth. There were no significant differences by smoking status in TH1 (IL-1β, IFN-γ) or TH2 (IL-4) class responses. Further investigation is required to determine whether these immune alterations play a role in development of adverse pregnancy outcomes associated with smoking. Unfortunately due to the nature of our urine cotinine assessment being that of a kit that detects presence of 200 ng/mL or more (positive) vs. not or less than 200 ng/mL (negative), our group is unable to generate a dose response curve. Nonetheless, our findings provide additional insight into the association of both decreased IL-10 with preterm birth as well as smoking with decreased IL-10, implicating smoking’s role in immune modulation that predisposed these women to a premature delivery.

While our data implicate deviation of IL1β, IL-10, and IL-6 in the pathogenesis of preterm birth, our study has a number of inherent limitations. Preterm birth is manifested by a myriad of underlying predispositions and environmental insults. Our cohort is predominantly African-American with low-education level and poor financial resources. Although the demographics limit generalization to all populations, our cohort is somewhat controlled. The uniformity of our sample also has the benefit of similar lifestyles, diet, and stress levels amongst participants. The variances demonstrated by our data shed light on the underlying immunologic differences predisposing a pregnant woman toward delivering preterm, especially in a cohort of inner city women at high risk.

Prior data have focused on cervicovaginal and amniotic fluid inflammatory milieu [1], [29], [30], [31], [32], [33], [34]. Cervicovaginal secretions are known to be colonized with bacteria whereas phlebotomy specimens are collected in a sterile fashion precluding degradation and change over time due to the influence of bacteria. Analyzing the secretions generated from the cervicovaginal mucosa undoubtedly facilitates evaluation of the innate immune cellular presence. A plethora of prior studies have shed light on immune variances as determined by cervicovaginal lavage. In 2018, Amabebe et al. published data on RANTES and IL-1B concentration in cervicovaginal fluid across two gestational age windows (20–22 6/7 weeks and 26–28 6/7 weeks GA) [1]. They demonstrated that increasing prevalence of vaginal anaerobes (e.g., bacteroides, fusobacterium, mobiluncus) over gestation corresponded with both increased risk for spontaneous preterm birth as well as IL-1B. Accordingly, FFN detection also corresponded with these changes in asymptomatic women to suggest predictive value from making such assessments.

Our project was designed to assess and analyze cytokine milieu via ELISA on phlebotomy specimens. Ex vivo analysis offers the important advantages of (1) ease of handling; (2) low or negligible risk on pregnancy outcome for the patient; (3) maintaining target cell types at their *in vivo* ratios with other cell types and non-cellular components; (4) retention of all blood components including serum; and, (5) ability to assess systemic inflammatory alterations in pregnancy that result from other portals of entry (e.g., periodontal disease, psychological stress, obesity, smoking/environmental respiratory insults, and cardiovascular inflammation from metabolic processes, etc.).

Our study design further adds to knowledge of immune modulation as expressed systemically and moreover longitudinally over the course of both gestations that result in uncomplicated term deliveries vs. otherwise uncomplicated spontaneous preterm deliveries. We additionally include data on how smoking affects longitudinal expression of a broad panel of cytokines over the course of gestation. Our group of investigators has experience in evaluating systemic inflammation in pregnancy and the Luminex assay methodology has been validated [12]. Furthermore, our group has previously published data on longitudinal modulation of immune system in otherwise uncomplicated pregnancies [5]. Data presented in this manuscript add to such findings in that we evaluated for variance in term vs. preterm deliveries longitudinally over the course of gestational as well as impact of smoking vs. no smoking over the course of gestation longitudinally. Both are areas that have been previously poorly understood as prior investigations have centered on singular time points of assessment such as upon presentation in preterm labor and/or shortly after delivery.

Our study has a number of other strengths. Both key clinical data and maternal biological samples were collected prospectively. Our group of includes statistical expertise as well as substantial experience in measurement and interpretation of cytokine levels using Luminex ELISA assays. Additionally, our study is unique in that we performed a longitudinal study in pregnant women on cytokine profiles across pregnancy ending in either uncomplicated term delivery or spontaneous preterm delivery.

Prior data demonstrate that IL-1β plays a key role in mediating the inflammatory response to bacterial vaginosis. Such response underpins the association of bacterial vaginosis with preterm birth. The response to the bacteria or lack thereof until inflammatory colonization sets in is likely rooted in polymorphisms in the host or gravid female’s immune system [23]. IL-1β is elevated in amniotic fluid during the midtrimester in women destined for preterm delivery [23], [24], [25]. Given this positive association, IL-1β has been labeled as a predictor for preterm birth. Midtrimester variance in IL-1β levels may be genetic as recent studies have elucidated polymorphisms in the IL-1β gene that lend toward preterm delivery [24].

Future studies in this area may prove useful in the care of the obstetrical patient by (1) identifying deviations from normal longitudinal patterns and (2) potentially identifying interventions to modify outcomes for at risk populations. Studies on the effect of interventions (e.g., progesterone whether vaginal, intramuscular, or oral; or, treatment of periodontal disease and pelvic infection) on cytokine trajectories may likewise shed light on mechanisms of action by known treatments for those at risk of delivering preterm.

Notably, the population studied is that of predominantly African-American community and may limit generalizability to other populations and their respective outcomes and immune responses in pregnancy. Given disparities in healthcare resources and research funding alike as they pertain to the underserved poor African-American population, our data are critical for the scientific and clinical community to add to the growing knowledge of our understanding of pregnancy biology in these underserved women. Better understanding of environmental triggers and underlying maternal immune response longitudinally over pregnancy may enable physician-scientists to identify modifiable variables that may ultimately impact pregnancy outcomes which are notably worse in the poor, underserved African American communities.

Analyses of cytokine trajectories associated with environmental insults like stress and smoking along with other adverse outcomes such as preeclampsia and stillbirth may be useful in better understanding mechanisms leading to their occurrence.
